# A practical approach for finding anti-debugging routines in the Arm-Linux using hardware tracing

**DOI:** 10.1038/s41598-024-65374-w

**Published:** 2024-06-26

**Authors:** Yeongung Park, Seokwoo Choi, Un Yeong Choi, Haimin Jin, Nurul Harzira Mohamad Nor, Yongsu Park

**Affiliations:** 1 The Affiliated Institute of ETRI, P.O. Box 1, Yuseong, Daejeon, 305-600 South Korea; 2 Department of Computer Science, Hanyang University, Wangshimriro 222, Seongdonggu, Seoul 0476 South Korea

**Keywords:** Computer science, Software

## Abstract

As IoT devices are being widely used, malicious code is increasingly appearing in Linux environments. Sophisticated Linux malware employs various evasive techniques to deter analysis. The embedded trace microcell (ETM) supported by modern Arm CPUs is a suitable hardware tracer for analyzing evasive malware because it is almost artifact-free and has negligible overhead. In this paper, we present an efficient method to automatically find debugger-detection routines using the ETM hardware tracer. The proposed scheme reconstructs the execution flow of the compiled binary code from ETM trace data. In addition, it automatically identifies and patches the debugger-detection routine by comparing two traces (with and without the debugger). The proposed method was implemented using the Ghidra plug-in program, which is one of the most widely used disassemblers. To verify its effectiveness, 15 debugger-detection techniques were investigated in the Arm-Linux environment to determine whether they could be detected. We also confirmed that our implementation works successfully for the popular malicious Mirai malware in Linux. Experiments were further conducted on 423 malware samples collected from the Internet, demonstrating that our implementation works well for real malware samples.

## Introduction

With the widespread use of embedded and Internet-of-Things (IoT) devices, malicious code is rapidly increasing in the Linux environment. To respond to this threat, considerable research efforts have been made to develop analytical techniques, including static and dynamic approaches, to defend against the threat of malicious applications.

Static analysis is an approach that analyzes the code without execution. The disadvantage of this method is that it cannot analyze encrypted code (also known as packed code) that is decrypted during execution or polymorphic code that changes its body during execution. Dynamic analysis is conducted while executing the code and has the disadvantage of making it difficult to analyze malicious code with an anti-debugging technique that detects or interferes with dynamic analysis tools, including debuggers.

Generally, malware uses various evasive techniques to deter analyses. For example, a malicious code can detect the presence of a debugger or hinder the attachment of a debugger. In addition, sophisticated malware, also known as evasive malware, can evade analysis by detecting artifacts in the execution environment or presence of analysis tools, thereby aborting execution or not performing any malicious activities upon detecting an abnormal status. Although the number of malware analysts is small, evasive malware is being developed daily. Hence, manually identifying and bypassing these techniques one at a time is very time-consuming and inefficient.

The advantage of using hardware tracing is that there is no speed degradation because the tracing work is performed at the hardware level in the CPU. In addition, it has the advantage of automatically avoiding debugger-detection techniques as there are almost no artifacts during tracing. These advantages have led researchers to propose hardware-assisted approaches that utilize Intel’s processor trace (PT)^1^ and the Arm embedded trace macrocell (ETM)^2^. With a hardware tracer, the execution flow trace can be quickly obtained with negligible artifacts.

In this paper, we present a practical approach for analyzing Linux malware, identifying debugger detection routines using ETM. Our objective was to obtain two traces (one without the debugger and the other with the debugger), thereby using ETM hardware tracing to determine trace divergence points for debugger-detection routines.

To achieve this, we reconstructed the execution flow of the compiled binary code from ETM trace data by comparing the disassembled code and memory map information, to resolve the addresses of the jump, call, and return instructions. To minimize the trace size, only the target Linux application process was traced using CPU isolation and cpuset in Linux. By comparing the two traces, the code chunk for debugger detection was automatically identified and patched.

The objective of this study was to answer the following research questions:

- *Research Question 1 (RQ1)*: In Arm-Linux environments, is it possible to trace malware using ETM without specialized devices?
- *Research Question 2 (RQ2)*: Is it possible to effectively detect various debugger-detection techniques in the Arm-Linux environments using hardware tracing?
- *Research Question 3 (RQ3)*: In real-world scenarios, can the proposed scheme effectively detect debugger-detection routines in evasive malware?
- *Research Question 4 (RQ4)*: Most debugger-detection techniques have been developed for x86-Linux environments. Do they work effectively in Arm-Linux environments?

To answer these questions, we conducted experiments, making the following contributions. The proposed method was implemented using the Ghidra plug-in^3^, which is one of the most widely used reverse-engineering tools for binary analysis. To verify its effectiveness, we implemented 15 debugger-detection techniques in the Arm-Linux environment, to test whether they could be detected. Our scheme was further tested on the Mirai^4^ malware, to demonstrate that our implementation works successfully. In addition, 423 Linux-malware samples were collected, conducting experiments to show that the proposed scheme works well on all executable samples having debugger detection routines.

The main contributions of this study in answering the research questions are as follows:

*(RQ1):* For Linux malware, fast, artifact-free control flow analysis tracing is possible using ETM hardware. The ETM trace can be obtained with negligible performance overhead. To this end, we developed an address resolver that reconstructs the execution flow of the compiled binary code from trace data for address space layout randomization (ASLR), as modern Linux uses ASLR for address randomization, which complicates the analysis.
*(RQ1):* To collect and analyze the ETM trace log, previous methods generating and analyzing long traces relied on specialized hardware and software, including Juno reference board^5^, DS-5^6^, and DSTREAM^7^. However, our approach does not require such dedicated hardware equipment and expensive software.
*(RQ1):* Unlike previous work, multi-threading and multiprogramming analysis are supported [Because extensive testing has not yet been conducted, supporting multi-threading and multiprogramming may not be fully funtional.]. We devised an effective process filtering method to trace only the target Linux application with ETM. This method uses CPU isolation and cpuset to allow the designated core to execute only the target Linux application while activating ETM. This method also improves the decoding performance because in the trace data, there is information only for the target application. Moreover, if the target application spawns a child process, the proposed method automatically keeps track of the child process employing the cpuset policy in the Linux kernel.
*(RQ4):* We analyzed and implemented 15 debugger-detection techniques for the Arm-Linux environment. In our experiments, the debugger-detection techniques worked correctly for nine out of 15 cases. After attaching gdb, the techniques worked correctly for seven out of 15 cases. (For comparison, the entire process was repeated for the x86-Linux environment, obtaining the same results.)
*(RQ2):* Upon testing, the proposed method successfully identified debugger-detection routines for all seven techniques (in the gdb-attach mode).
*(RQ3):* In addition, a case study is included for testing against Mirai^4^ malware, which is one of the most popular malware families that can detect anti-debugging routines in Linux. The experiments were conducted on 423 Linux malware samples collected from the Internet, thereby manually determining that 57 of them had debugger detection routines. Among them, seven samples were successfully executed in our experimental environments. The implementation worked correctly for all seven samples having debugger-detection routines.

The remainder of this paper is organized as follows: The background is explained in the “Background and Challenges” section. In the “Proposed method” section, the proposed scheme that uses a hardware tracer to trace the Linux malware application is described. The results of evaluating the scheme are presented in the “Evaluation” section. Several issues are expounded upon in the “Discussion” section, and related work is summarized in the “Related work” section. Finally, conclusions are drawn in the last section.

## Background and challenges

First, the various debugger-detection techniques for Linux systems are explained, subsequently illustrating how the hardware tracer on the Arm platform works and how the decoder processes the trace data. Finally, the challenges of tracing Linux applications using a hardware tracer are described.

### Debugger-detection techniques for the Linux system

Debugger detection routines are used to determine whether a program is under debugger control. These routines are commonly used for software protection to prevent unauthorized reverse engineering, debugging, or modification of program behavior as well as to prevent malicious users or researchers from analyzing the inner workings of the software, particularly when handling sensitive data and security-related applications.

Generally, malware uses various debugger detection techniques to deter analyses. If malicious code detects the presence of the debugger, it aborts execution or does not perform any malicious activities. Hence, quickly finding and bypassing these techniques are crucial in reducing the time and effort required for malware analysis.

The various debugger detection techniques surveyed are briefly explained as follows:

$\textcircled {1}$ *Debugger detection using ptrace()* This is one of the most widely used techniques for checking the presence of debuggers. ptrace() provides functionalities for accessing the code, data, stack, heap, and registers of the target process. In other words, the address space of the target process can be accessed at the user level. Using this property, someone can attach to the process and modify, analyze, and debug the memory content. Because ptrace() provides such powerful functions, it can also be used for effective debugger detection. A less than zero result of ptrace(PTRACE_TRACEME,0) indicates that debugging is already in progress.

$\textcircled {2}$ *Hiding call to ptrace()*^8^ If ptrace() is in a location that can be easily found in the source or binary code, analysts can easily locate the relevant anti-debugging routines. Here, we describe a method for hiding the ptrace() call using dynamic loading.

Dynamic loading refers to the concept of loading a library and retrieving the library function addresses during runtime. As address resolution is performed at runtime, ptrace() does not appear when the code is disassembled before execution. To achieve this, we need to call dlopen() to load the library and dlsym() to resolve the function address of ptrace(). The dlsym() function returns the address corresponding to the symbol according to the dynamic link library, allowing the addresses of the function and variable to be obtained at runtime.

$\textcircled {3}$ *Debugger detection using SIGILL* The SIGILL signal occurs when an illegal instruction is executed and can be used to detect debuggers. In Arm-Linux environments, the udf instruction is an invalid opcode instruction. The execution of udf generates SIGILL, and the corresponding handler is invoked. If the debugger detects this signal and handles SIGILL, the original handler is not executed. Tools such as Pangu^9^ use this technique for debugger detection.

$\textcircled {4}$ *Debugger detection using SIGSEV*^9^ The SIGSEGV signal occurs when there is a segmentation fault. This technique detects debuggers by invoking SIGSEGV and checks the behavior. In normal execution, when SIGSEGV occurs, the corresponding handler is invoked. However, in debugging environments, if the debugger intercepts SIGSEGV, the original handler may not be called. The technique checks this difference.

$\textcircled {5}$ *Debugger detection using SIGTRAP*^9^ In Linux, the SIGTRAP signal is used for debugging. If this signal is present, the debugger intercepts the execution, and the originally registered handler may not be executed.

$\textcircled {6}$ *Debugger detection using environment variables* This technique detects debuggers by checking the environmental variables created while running the debugger. For example, when gdb is executed, the environment variables ‘COLUMNS’ (terminal width length), ‘LINES’ (terminal height length), and ‘_=gdb_path’  (the path of gdb) are set. Hence, any of these three environmental variables being set, indicates that the debugger is present during execution.

$\textcircled {7}$ *Debugger detection using file descriptors* When a program is executed in Linux, fixed file descriptors of 0 (standard input), 1 (standard output), and 2 (standard error) are assigned. Therefore, when a file is opened for the first time, in an ordinary case, 3 is assigned to the file descriptor of the new file.

However, when opening a file for the first time in the gdb, because more file descriptors have already been used for gdb-related internal work, a number greater than 3 is assigned to the file descriptor, to allow gdb to be detected.

$$\textcircled {8}$$ *Debugger detection using /proc/$PPID/cmdline* /proc/$PPID/cmdline contains the contents of the command line used when executing the target process. If /proc/$PPID/cmdline contains ‘gdb,’ this is regarded as indicating the presence of the debugger.

$$\textcircled {9}$$ *Debugger detection using /proc/$PPID/status* The file /proc/$PPID/status contains diverse information on the execution status of the parent process. If it contains ’gdb’ or ’ltrace,’ the debugger is running.

$\textcircled {10}$ *Detecting breakpoints* This technique detects the debugger by reading the opcode of the program and stopping execution if it matches the opcode of the breakpoint used by the debugger. The opcode of the breakpoint instruction in AArch64 environments is 0xd4200000 (BRK), which can be checked in the code region.

$\textcircled {11}$ *Debugger detection using LD_PRELOAD* If the LD_PRELOAD environment variable is set, during runtime, the dynamic library is loaded and executed from this environment variable value. Sometimes the user of gdb uses LD_PRELOAD to attach a custom library during debugging. If we detect that the content of LD_PRELOAD is changed during runtime, we can regard that debugging is underway.

$\textcircled {12}$ *Debugger detection by checking /proc/self/status* This technique checks whether debugging is being performed by examining the execution status of the program and checking the contents of the /proc/self/status. When a program changes from a non-debugging to a debugging state, the TracerPid entry, which is the process id of the debugger in /proc/self/status, also changes from zero to a nonzero value. Therefore, a debugging in progress can be determined by checking the TracerPid value in the status file.

$\textcircled {13}$ *Debugger detection by checking /proc/self/fd* This technique detects debuggers by checking the number of file descriptors owned by the process. When initiated, gdb forks a process for debugging. When performing forking, the file descriptor (fd) owned by the parent process is inherited by the child process. Because gdb often opens multiple files, the file descriptors of them are also inherited by the target process. Specifically, the number of file descriptors owned by the process is in /proc/self/fd, and this file can be checked. As this file is a directory file, each line corresponds to file information in this directory. For a new process, it contains six lines for the six files (‘. /, ../, 0, 1, 2, and 3’). If the number of lines is greater than six, we regard this as the presence of debuggers.

$\textcircled {14}$ *Detection before main()* If we hide the debugger detection routine in the not-so-easily visible region in the code, the security can be increased. For example, if we use the “constructor” attribute in the C code, we can call the function before main(). For example, if we declare the function as follows, “void __attribute__((constructor)) before_main_function() {...}”, this function is called before main()^8^.

$\textcircled {15}$ *Detection by checking execution speed* If the target program is executed using debuggers, the execution speed is slower than normal. An execution speed that is slower than the threshold indicates that the program is being debugged.

### Hardware tracing on Arm platform

The Arm platform provides CoreSight, a comprehensive system with invasive and noninvasive hardware-based debugging features. ETM^2^ is CoreSight’s noninvasive debug module, which is attached next to the CPU core and records all instructions and data accesses. Trace information from ETM is called a trace packet and the ETM contains approximately 30 types of trace packets. We briefly describe the major packets: A-sync, Context ID, Branch addresses, and Atoms relevant to our study. For other packet types, the reader is referred to the embedded trace macrocell architecture specification^2^.

1. A-sync packet (Alignment synchronization packet): This packet is used for alignment synchronization. Whenever the ETM is enabled, the first packet output is an A-sync packet, and periodically, ETM outputs an A-sync packet.
2. Context ID packet: Context ID is related to the process/thread ID, which is managed by the operating system. When the Context ID changes, the ETM outputs a Context ID packet, providing a new value.
3. Branch address packet: this packet indicates that there is a change in the program flow. It contains the branch address. This packet can be generated by an exception, an (indirect) branch including return.
4. Atom packet: This packet is related to a conditional branch. There are two types of atoms: E and N. The E atom indicates that the conditional branch is taken, whereas the N atom represents not taken.

**Figure 1:**
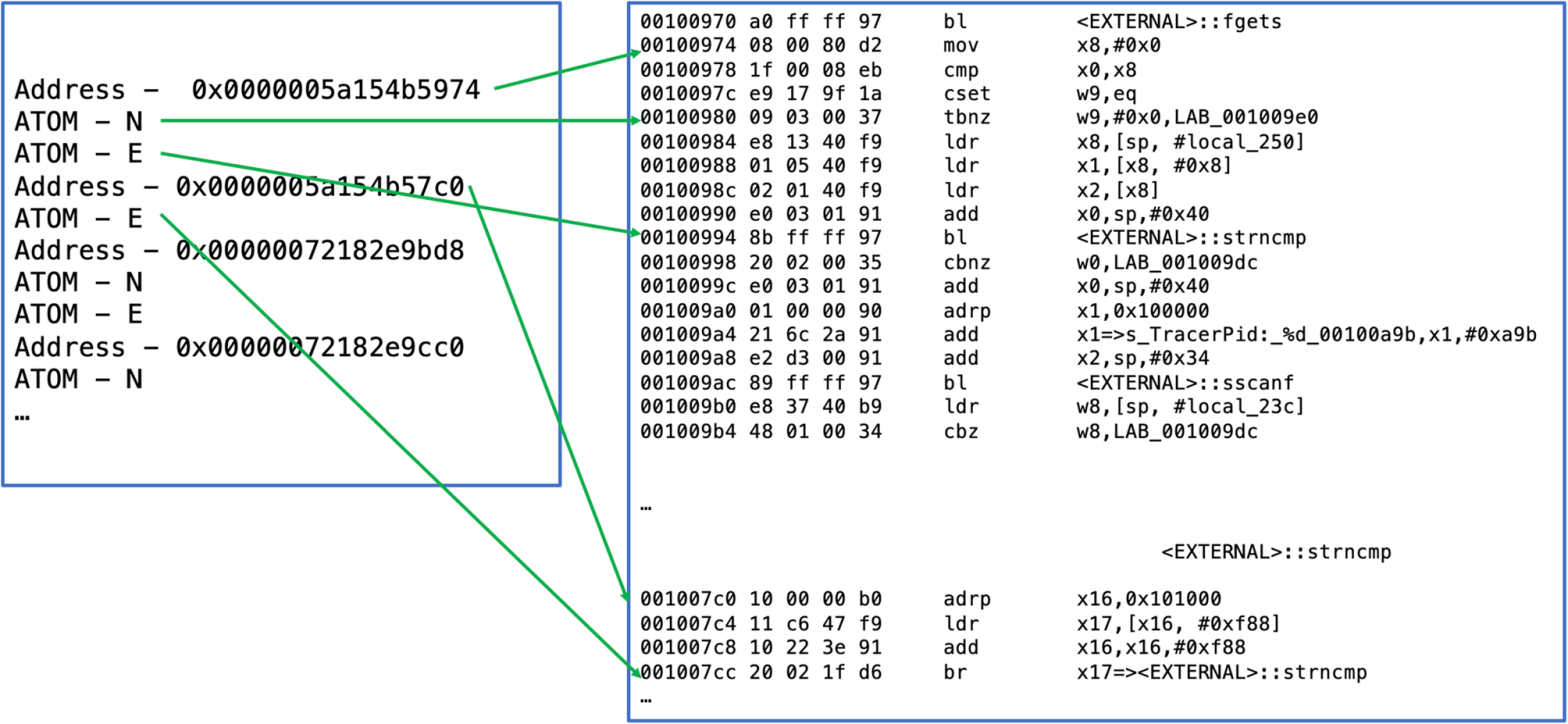
Example of log chunk for the hardware trace and corresponding assembly code chunk.

Next, the interpretation of ETM trace logs is briefly explained. Figure 1 shows an example of ETM trace log and the corresponding assembly code. On the left-hand side, the first line ‘Address - 0x0000005a154b5974’ is the branch address packet, which corresponds to the second line in the assembly code: ‘00100974 08 00 80 d2 mov x8, #x0’, which implies that this instruction was executed. The second line of the trace is the Atom packet, with ‘N’ indicating that the conditional branch was not taken. In the assembly code, starting with the address 0x00100974, the first conditional branch is ‘00100980 09 03 00 37 tbnz w9, # 0x0, LAB_001009e0.’ Because this branch was not taken, the control flow moved to the next instruction at 0x100984. The third line in the trace is ‘ATOM - E’ implying that the next conditional branch was taken. The corresponding branch instruction is ‘00100994 8b ff ff 97 bl <EXTERNAL>:: strcmp.’ This branch was taken, and the execution flow moved to the address 0x001007c0. In this manner, the execution flow can be reconstructed from ETM trace logs.

Because ETM records all the instructions executed from a hardware perspective, it does not account for the process or thread context from a software perspective. For this reason, Arm CoreSight provides comparators that control the activation of ETM to support selective tracing. For example, an address-range comparator activates the ETM only when the core executes instructions in a specific address range. CoreSight also supports other comparators such as a single-address comparator, context ID comparator, and virtual machine ID comparator. The context ID comparator can be used to record only the instructions in the desired process.

Arm CoreSight provides an embedded trace buffer (ETB), which is an on-chip trace buffer of size 64 kB, storing trace data from ETM. ETB is a circular trace buffer shared by ETMs in each core. In addition to ETB, Arm CoreSight provides an embedded trace router (ETR) to support a large trace buffer. This is because the buffer size of ETB is small to store a large amount of trace data from a long-running program. ETM generates an overflow packet when the trace buffer is full, indicating that trace data may be lost due to trace buffer overflow. However, CoreSight does not provide a function for stopping the core or preventing overflow. The ETR stores trace data in the system RAM through direct memory access (DMA) and can access only physically contiguous memory spaces in the primary mode. The allocation of physically contiguous memory spaces may be limited to embedded devices. Considering this limitation, ETR provides a scatter-gather mode that receives the page table as input and supports non-contiguous memory space. A gigabyte-sized trace buffer can be allocated by using the scatter-gather mode of the ETR.

### Decoding trace packets

A trace packet from ETM consists of a 1-byte header and *n*-byte payload. The header byte indicates the type of trace packet, and the payload contains the trace information. The payload also contains a bit field indicating whether the trace packet contains specific information, as detailed in the specifications. Based on the bit field, a trace packet may not contain specific trace information, and the type and bit field determine the length of the trace packet. Therefore, the decoder must sequentially decode the trace packets to determine the exact start position of the next packet. For example, an ‘Address with Context instruction trace packet’ has a bit field indicating whether it includes a context ID or a virtual machine ID. The bit field determines the exact length of the ‘Address with Context instruction trace packet.’ The decoder can identify the exact start point of the subsequent trace packet by finding the header of the ‘Address with Context instruction trace packet’ and reading the bit field of the payload to know the actual size of the packet. Similar to previous studies^10,11^, we used an open-source analyzer, ptm2human^12^, to convert streams into readable format.

### Address resolving of Linux application

The ETM provides Branch address and Atom packets as the main trace packets for reconstructing the program execution flow. Therefore, the trace analyzer must understand the control flow and the address of each instruction in the target process.

As shown in the example above, the address recorded by ETM is different from that in the code disassembled from the executable file. In addition, the conditional branch instruction should be correctly interpreted for each Atom packet.

### Challenges

In this subsection, we describe the major challenges encountered when hardware tracing is used to analyze Linux applications, including malware.

*Selective tracing.* Using the comparators of the ETM cannot selectively trace the target Linux application. The context ID comparator determines ETM activation by comparing the value of the CONTEXT_IDR register, which stores the thread ID when the task changes by scheduling in the Arm-Linux kernel. This means that one thread can be tracked with one slot of the context ID comparator; however, many Linux applications have multiple threads, and the number of threads to be created cannot be determined while running a Linux application. Furthermore, the number of comparators provided by CoreSight is eight in ETMv4.

Let us suppose that we filter only the tasks belonging to the target Linux application process using the context ID comparator. In this case, the values of the context ID comparator should be managed while tracking thread creation and exit, which is inefficient and poses a high risk of trace data loss. Alternatively, only the threads of the target application can be collected during decoding after recording the entire execution of all processes. However, this method must decode unnecessary trace data, which significantly increases the decoding time. Decoding results in the highest performance overhead in all steps when utilizing the hardware tracer. Consequently, we must ensure that the ETM records only the target Linux application process for a selective trace.

*Continuous tracing.* Although CoreSight provides the ETR and scatter-gather modes to support large trace buffers, trace data can still be lost. This is because, in certain cases, complex Linux applications can generate trace data exceeding 10 GB. Even if the scatter-gather mode of ETR supports a large trace buffer, allocating more than 10 GB of memory is difficult in mobile or embedded devices. Furthermore, while extracting massive trace data from the trace buffer, the core executes numerous instructions, and the ETM generates hundreds of megabytes of trace data. This is because CoreSight does not provide a feature for stopping the core when the trace buffer is full. We manually set the ETB buffer size to be maximum to support the acquisition of large trace data.

*Resolving addresses in ASLRed Linux.* The ETM trace log contains minimal information to minimize the log size, requiring inference when analyzing program execution from the log. For example, hardware tracers, such as ETM and Intel-PT provide 1-bit information to indicate whether a branch is taken. To correctly interpret this bit, the previously executed address information should be used to find the first branch instruction that is reachable from this address. Because the ETM does not explicitly generate all the executed addresses, an interprocedural control flow graph (ICFG) is required to analyze the target program using the trace data.

In addition, we need to know the address of the loaded binary code in advance to reconstruct the execution flow from the trace data. Modern Linux systems use ASLR, which implies that for every execution, the loaded address for the code is different for the same program.

## Proposed method

**Figure 2:**
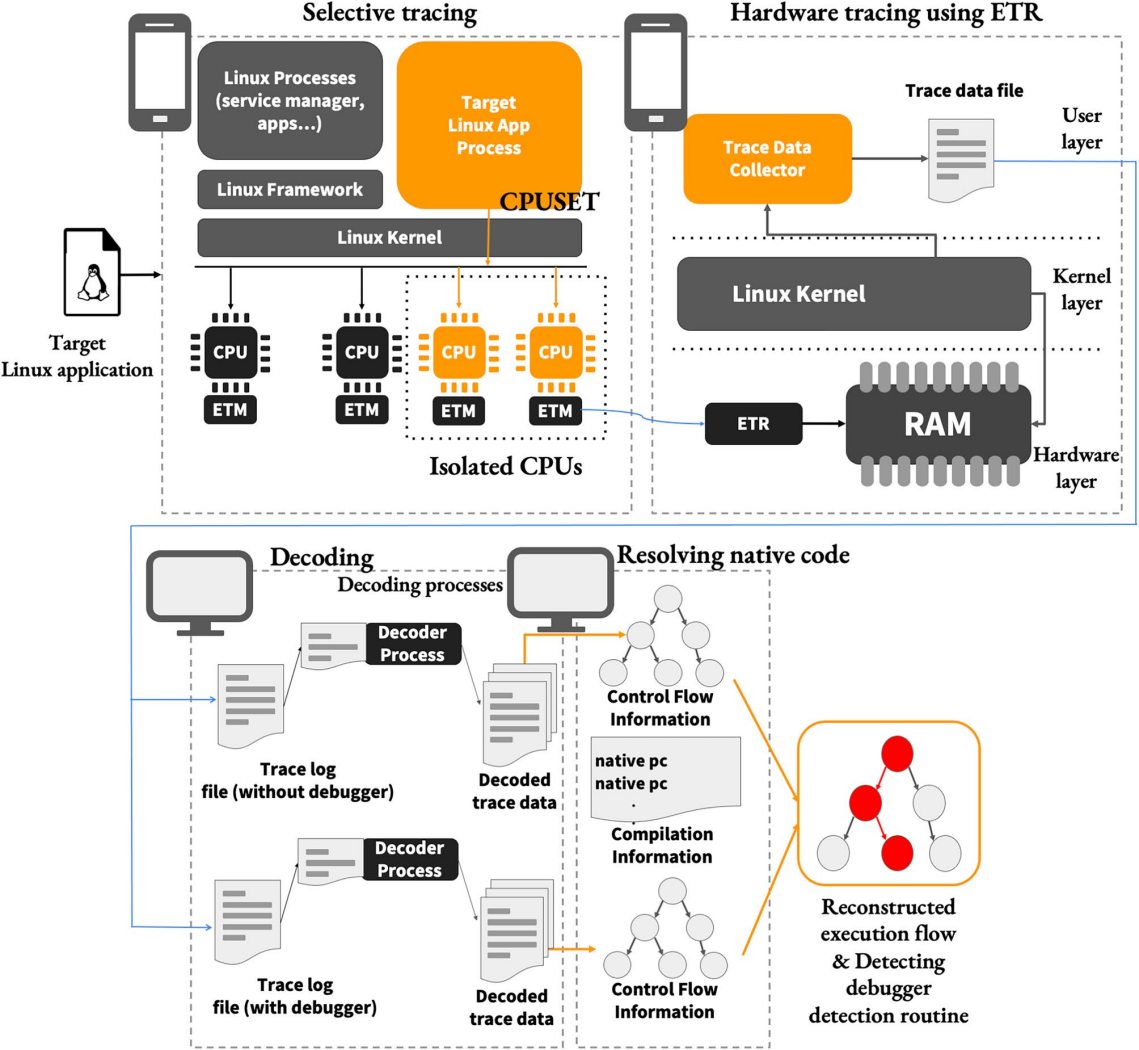
Overview of the proposed method.

In this section, the proposed method for determining anti-debugging routines is described. In addition, this section describes detailed solutions to the challenges mentioned earlier.

### Overview

The proposed approach first aims to reconstruct the execution path of the compiled and optimized native code in the trace data from ETM. To achieve this, first ETR is set to scatter-gather mode to allocate a gigabyte-sized trace buffer. As the Linux kernel does not allocate tasks to isolated CPUs, the interrupts and noise trace data are not generated. Next, the userland process is run, to collect trace data from ETR and store them in a file. The target Linux application is now ready to run.

Once the ETM and trace buffer are ready, the target Linux application is installed and executed. By configuring *cgroup* and *cpuset* to allow the target Linux application to run only on isolated CPUs, we can selectively trace only the target Linux application using ETM. Once obtained, the trace data are decoded to generate human-readable data. Then, the execution flow of the compiled native code is reconstructed using the decoded trace data. To this end, the addresses of the binary code are interpreted using memory-map information.

Finally, another trace is obtained from the target program using the debugger. After reconstructing the execution flow, the two types of flow information are compared to determine the debugger detection routine. The overall procedure is illustrated in Fig. 2.

### Selective tracing

Selective tracing uses ETM to trace only the target process. To achieve this objective, the CPU is allowed to execute only a specific process on a specific core. In other words, selective tracing ensures that only the target Linux application’s process runs on a specific core. CPU isolation and selective resource allocation are used to achieve selective tracing so that ETM is enabled and the isolated CPU core runs only the target Linux application.

#### Isolating CPU

Isolating the CPU causes the designated CPUs to wait without running anything except the desired process. The Linux kernel provides a kernel boot argument called *isolcpus* which excludes the specified CPUs from the Linux scheduling target. On the upper-left side of Fig. 2, selective tracing shows the isolating CPU. As shown in Fig. 2, the Linux kernel manages the isolated CPUs, ensuring that they do not execute any code (except for the target program), including background processes. Even when the ETM is activated on an isolated CPU, no trace data are generated in the trace buffer until the target program begins to execute. To run a specific process that we want to trace on an isolated CPU, the process is run by explicitly specifying the CPU affinity using related API function calls.

Isolating the CPU is valuable when using an ETM. Activating the ETM on a non-isolated CPU may also generate trace data of unwanted processes that may need to be filtered out during the decoding process. Alternatively, the processes running on the CPU must be filtered while changing the value of CoreSight’s comparator at the exact timing. However, these filtering methods incur unnecessary performance loads. Because the ETM is activated on an isolated CPU and selectively runs only the target processes, the trace buffer holds only the trace data from the target Linux application. This also reduces the decoding cost as the decoder decodes only the essential trace data by selective tracing.

#### Selective resource allocation

Selective resource allocation ensures that only the target Linux application executes on a specific CPU. The Linux kernel provides the *cgroup* module, which controls resource allocation for process groups in task units. The allocation of resources to a specific process group can be controlled using diverse subsystems of the *cgroup*, such as *cpuacct*, *cpuset*, *memory*, and *devices*. We used *cpuset* to ensure that the target Linux application ran only on an isolated CPU. *cpuset* is a subsystem that binds individual CPUs and memory nodes to *cgroups* and allows a specified process group to be scheduled only on specific CPUs. Linux defines various *cgroup*s such as *top-app*, *background*, and *system*. *Top-app* is the *cgroup* setting applied to the application currently displayed on the screen. That is, the settings of the *top-app cgroup* are applied to the application that is being run. Therefore, by adjusting the *cpuset* settings of the *top-app*, we can control the CPU group to execute the application currently displayed on the screen.

Figure 2 shows the usage of *cpuset* in selective tracing. ETM is pre-enabled on the isolated CPU. Because the target process that will run on the isolated CPU is not explicitly specified, no process runs on the isolated CPU at this point; therefore, the ETM does not generate unnecessary trace data. Before running the target Linux application, the *cpuset* of the *top-app cgroup* is set to isolated CPUs. Then, as shown in Fig. 2, using the *top-app* setting, the target Linux application is executed only on the ETM-enabled isolated CPUs, and only the execution information of the target Linux application is recorded in the trace buffer.

The *top-app cgroup* also allows the child process of the target Linux application to run only on the isolated CPU. We have frequently observed that Linux applications, including malware, create child processes at runtime. Malicious applications create child processes for various purposes, such as executing script files and running binary files. Therefore, the execution of the child processes must be recorded to trace the overall execution of the target Linux application. We used *cgroups* to selectively trace the entire execution of the target application by tracing not only the target Linux application but also its child processes.

#### Allocating maximal size for the ETR buffer

The ETR is used to store the trace data from ETM in the system memory, as shown in Fig. 2, by allocating a sufficiently large trace buffer. Because the system memory generally has more space than the on-chip buffer such as ETB, the trace buffer space can be amply used by utilizing the ETR. However, when ETR in the primary mode uses the system memory as a trace buffer, the trace buffer accessed by ETR must be physically contiguous because ETR in the primary mode only takes the base address of the trace buffer and its size as arguments. For example, if 100 MB of trace buffer is to be allocated to the system memory, the Linux kernel must support 100 MB of physically contiguous memory, which may not be supported by typical mobile or embedded devices.

CoreSight supports the scatter-gather mode for the use of a non-contiguous memory space as a trace buffer. ETR in the scatter-gather mode takes a page table as argument and accesses the fragmented memory space represented by the page table as one memory space. In general, mobile devices support the allocation of non-contiguous physical memory space well. We configured ETR for the scatter-gather mode and allocated 1-2 GB of trace buffer, further adjusting the event count value based on the trace buffer size to minimize the occurrence of interrupts.

### Resolving addresses in native code

This section describes the reconstruction of the execution flow from the hardware traces obtained when executing the binary code compiled in C/C++. First, we explain how to obtain addresses for branches, interpret addresses, and reconstruct the execution flow. Finally, we explain how to find the location of the debugger detection routine using the difference between the execution traces with and without the attached debugger.

#### Interpreting packets to get relevant addresses

After obtaining the ETM trace, the trace files should be decoded. Although the decoded trace consists of diverse packets, we focused on the following packets: TraceOn, Context ID, Atom, Branch address, and Exception.

Among these, the TraceOn packet occurs sporadically after the start of program execution, as tracing sometimes temporarily stops and then resumes. No special processing is required.

The Context ID packet contains details about the execution environment, including the thread ID, which is necessary for interpreting the execution flow. Once this packet appears, it is interpreted as the execution of the same thread until the thread ID changes in the next Context ID packet.

The Branch address packet contains a branch address, which is a virtual memory address. Because the Linux system uses ASLR by default, even if the same application is rerun, all regional and function addresses are changed. Hence, the function and code chunk this address value represents should be interpreted, as explained in detail in the next section.

The Atom packet has the value E in the case of a conditional jump and value N if no such jump occurs, implying that the next instruction is executed.

In the case of an E value, the address it jumped to is determined by examining the corresponding instruction. To determine the next instruction corresponding to the N value, we must know the instruction that is currently being processed.

If the ETM.ReturnStack functionality is enabled, an Atom-E packet is generated whenever the function returns, that is, when the ret instruction is executed. The previous call history is managed in the form of a stack, and when the ret instruction is executed, it is interpreted as returning to the next instruction of the call (bl) instruction.

Exception packets are generated when an exception such as an interrupt occurs. Among various interrupts, timer interrupts can occur during execution. When interpreting the address value of the interrupt, if it belongs to the kernel or system, a system/kernel-related handler is executed. Therefore, we ignore exception packets to trace the execution flow.

#### Address matching

**Figure 3:**
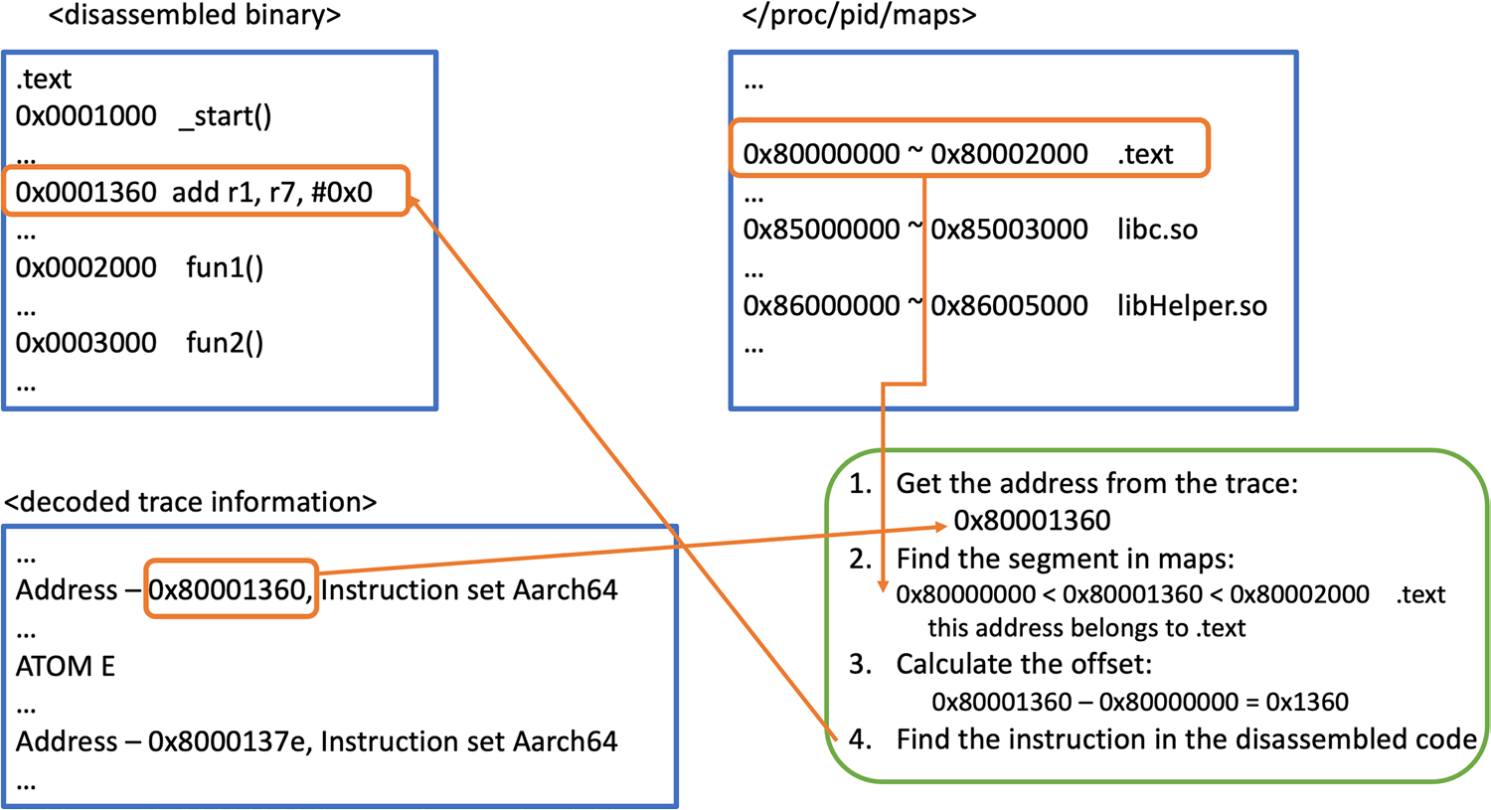
Procedure for determining the instruction from the trace address.

The trace packets were obtained through ETM, where they contain the addresses. Because Linux systems use ASLR, address matching must be performed to interpret the address values in the trace data, as detailed in the following steps. An example of this procedure is shown in Fig. 3.

1. *Disassembling the target binary code*: The target code is disassembled using a disassembler running on Linux. Although traditionally objdump is most often used as a disassembler, we used Ghidra^3^ to perform the disassembling task. Ghidra is an open-sourced disassembler developed by the NSA and is one of the most widely used reversing tools in binary code analysis for accuracy and usability. Ghidra provides free access to disassembled information through plug-in programs. The upper-left part of Fig. 3 shows an example of disassembled code.
2. *Obtaining memory mapping information while executing the target binary code*: In Linux, the /proc/PID/maps file contains memory segment information on mapped areas such as executable code segments (code execution areas), data segments, stacks, and dynamic libraries. This information is obtained when executing the target program to reconstruct the memory content. The upper-right part of Fig. 3 shows an example of /proc/PID/maps.
3. *Trace address interpretation*: From the information obtained from Steps 1 and 2, the trace address is calculated as follows. First, the address of the trace is compared with the segment information in /proc/PID/maps. If it belongs to a segment of interest (i.e., .text, .so, .a areas), we reconstruct the memory address information to identify the code chunk of the executed function. To this end, the start address of the corresponding segment in /proc/PID/maps is subtracted from the trace address to obtain the reconstructed address. Then, from this address, the corresponding instruction in the disassembled binary is determined. The lower-right part of Fig. 3 shows an example of this procedure. Whether the reconstructed address belongs to the function body in disassembled information can be further checked. All the addresses in the ETM trace can be interpreted in the same way, whereby the reconstructed address value of the trace is used to interpret the function that is called and the code chunk it jumps to. This simple calculation is possible because the Linux loader loads the code in the relevant area contiguously. Through this task, the memory address of the executed program can be reconstructed by functions and code chunks.

#### Reconstructing the control flow trace

In analyzing the trace of the Linux target program, a large amount of initialization code is executed before the program start point (. start() / _start() function in the. text area). This execution-related information appears at the beginning of the trace log and can be skipped before the starting point.

In the disassembler, a virtual pointer is placed at the start position of the _start()/. start() function ($=ptr_2$ in Algorithm 1), and the corresponding packet that has the same address is searched in the trace file (= $ptr_1$ in Algorithm 1). We now proceed with the interpretation from this point in the trace file.

If a Context ID packet is found, the thread information can be obtained, whereas finding a Branch address packet indicates that there is a jump to that address, thereby incrementing the virtual pointer and attempting to match it with the nearest jmp/call instruction. If the target address value of this instruction matches that of the Branch address packet, the virtual pointer is moved to the target address value of the instruction. For the call (br) instruction, the calling address is pushed to the virtual call stack.

If an Atom packet is found, whether the packet content is N or E should be checked. If it is N, since the branch is not taken, the virtual pointer is incremented in increments of one until a conditional jump instruction is found among the subsequent instructions. Because no jumps occurred in this instruction, the virtual pointer is incremented by one. If it is E , there are two cases: conditional jumps or returns. Thus, the virtual pointer is incremented by one to search for a conditional jump or a return among subsequent instructions, whereby the first instruction found is the corresponding instruction. In the case of a conditional jump, a virtual pointer is placed at the jump address. In the case of a return, the virtual pointer is placed at the next instruction of the address obtained by popping from the call stack.

Thus, the control flow can be reconstructed. We implemented this as a Ghidra plugin that highlights the executed code to facilitate the examination of executed instructions. A complete description of the proposed method is presented in Algorithm 1.

**Algorithm 1:**
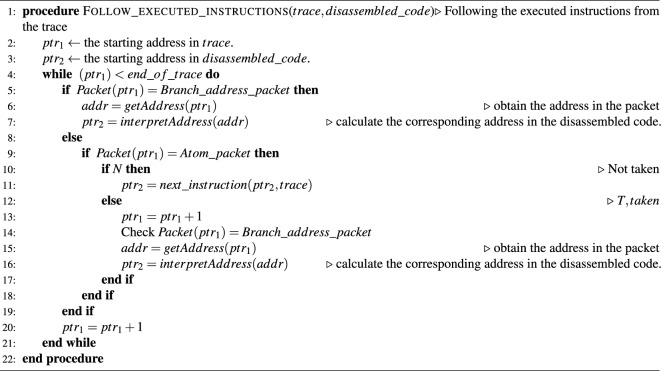
Reconstructing the control flow from the trace

#### Finding a fork in two traces to detect the debugger-detection routine

The procedure for determining the part of the code containing the debugger detection routine is as follows.

1. *Running the target program to obtain the trace (no debugger)*: First, the target program is run without attaching any debuggers, and the program execution trace data are collected. This provides a baseline for the control flow of the program without debugger intervention.
2. *Running the target program to obtain another trace (debugger attached)*: The same program is rerun but this time with a debugger attached during execution, and execution trace data are collected. This allows us to observe changes in the behavior of the program when a debugger is present.
3. *Analyzing two traces to find differences in the control flow*: Algorithm 2 was used to analyze two traces to find the differences in control flow. If a difference is detected, we regard this point as a candidate for the debugger-detection routine. A fork reoccurring after merging the flow also becomes a candidate for the debugger detection routine.
4. *Identifying debugger detection routines*: After finding all candidates in this way, the code is manually searched to find debugger-detection routines.
5. *Patching:* Once the debugger detection routine is successfully identified, patching is performed as follows: First, the fork occurring due to the conditional jump instruction is determined. This instruction is then patched with either NOP or an unconditional jump. (The user should manually check other instances and patch each instance individually.)

**Figure 4:**
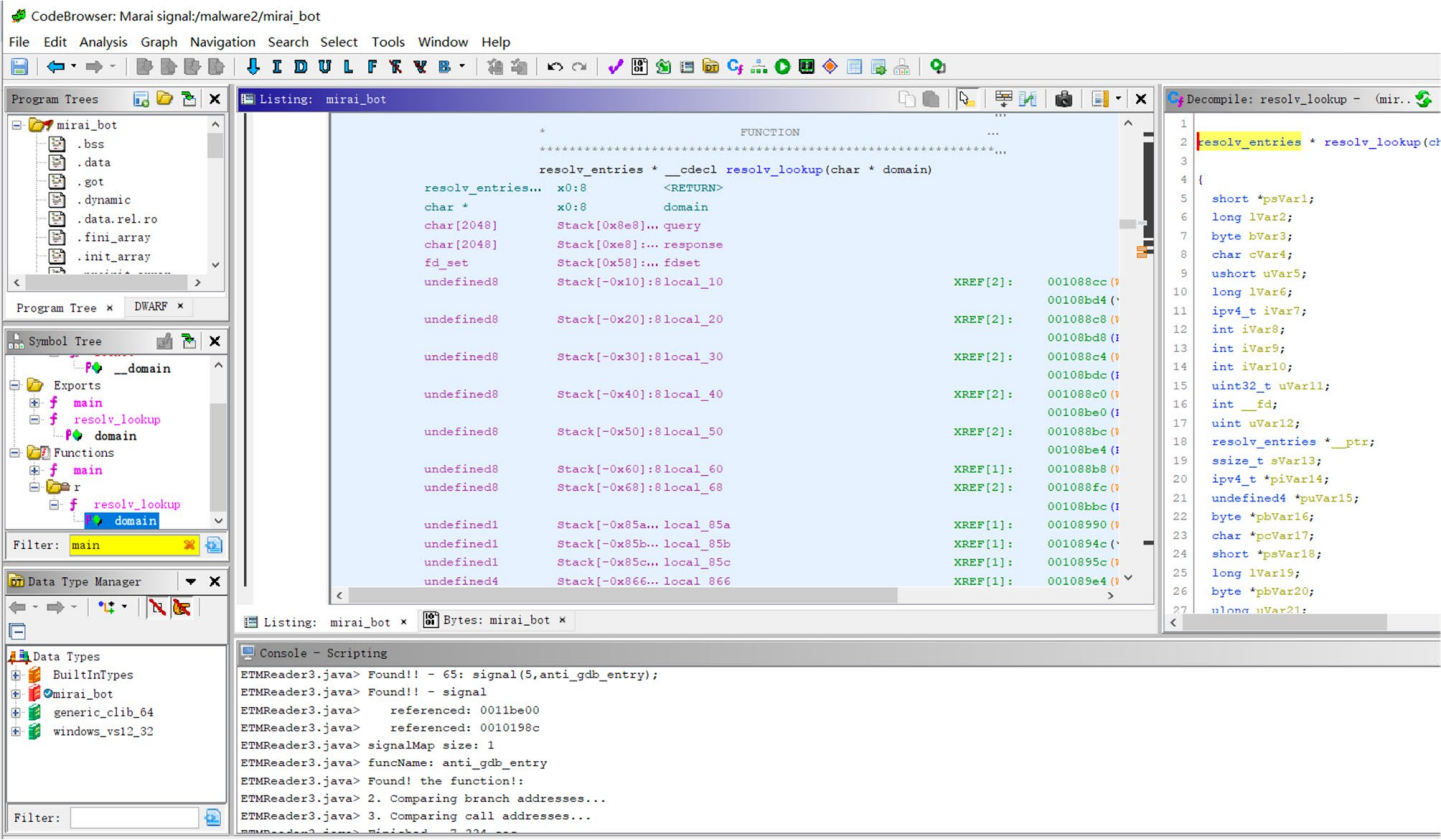
An example for detecting the debugger detection routine using the proposed scheme.

Figure 4 shows our experimental results where it automatically detects the debugger-detection routine and then automatically patches the conditional jump to bypass checking.

As described in the next section, 15 debugger detection techniques were implemented, conducting experiments to test whether this approach works for the Linux system. In addition, practical issues and methods for detecting the debuggers are discussed.

However, dealing with complex real examples is challenging because forks and trace merges can occur for various complicated reasons, leading to a number of false positives. Thus, a method to reduce them is required.

Hence, we used a heuristic method to minimize the number of candidates. Each debugger-detection routine has specific behaviors , such as using specific application programming interface (API) function calls or invoking / handling exceptions. If no patterns were observed, the case was excluded from the candidate list. In the next section, real examples are analyzed to explain how to reduce this problem as much as possible.

**Algorithm 2:**
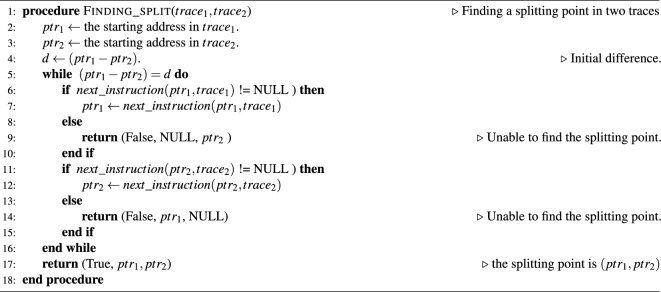
Finding a splitting point in two traces

## Evaluation

Recall that in the Background section, we summarized the 15 debugger detection methods that we surveyed in Arm-Linux environments. Each technique was implemented and tested to ensure that it was working correctly, further testing to determine whether automatic detection was possible using the proposed technique. The results are summarized in Table 1.

Additionally, as a real-world application, the Mirai^4^ malware, which is one of the most prevalent malware on Linux was selected to test whether our scheme can detect the debugger-detection routine in this sample. In addition, 423 real malware samples were collected to conduct experiments.

### Implementation and experimental settings

Two execution environments: Arm (AArch64)-Linux with debugger and Arm (AArch644)-Linux without debugger were configured. When using the debugger, we have two options: execute the target program within the debugger or attach the debugger to the running target program. In most cases, these two options yielded the same results; however, in some cases, they differed, as shown in Table 1.

For Arm Linux, we used the DragonBoard 845c (db845c) board in Linux Version 4.19. The host PC was a Linux-based Xeon E5-2680 with 128GB RAM workstation.

**Table 1:** Experimental results for each debugger-detection technique.

Name	AArch64-Linux	AArch64-Linux (attach)	Automatic-detection
$\textcircled {1}$ Detection using ptrace()	O	O	O
$\textcircled {2}$ Hiding call to ptrace()	O	O	O
$\textcircled {3}$ Detection using SIGILL	X	X	–
$\textcircled {4}$ Detection using SIGSEV	X	X	–
$\textcircled {5}$ Detection using SIGTRAP	O	O	O
$\textcircled {6}$ Detection using environment variables	X	X	–
$\textcircled {7}$ Detection by checking the file descriptor	X	X	–
$\textcircled {8}$ Detection by checking /proc/ppid/cmdline	O	X	–
$\textcircled {9}$ Detection by checking /proc/ppid/status	O	X	–
$\textcircled {10}$ Detection by checking breakpoints	O	O	O
$\textcircled {11}$ Detection by checking LD_PRELOAD	X	X	–
$\textcircled {12}$ Detection by checking /proc/self/status	O	O	O
$\textcircled {13}$ Detection by checking /proc/self/fd	X	X	–
$\textcircled {14}$ Detection before main()	O	O	O
$\textcircled {15}$ Detection by checking execution speed	O	O	O

O, working correctly; X, not working or working incorrectly; –, not tested.

### Experimental results for anti-debugging techniques

Anti-debugging techniques can be broadly classified into debugger detection, debugger attachment prevention, dump generation prevention, anti-disassembling, and code obfuscation techniques. To the best of our knowledge, we have surveyed all the debugger detection techniques applicable to the Linux environment and presented them in the Background. The issues encountered in the implementation of all 15 debugger detection techniques are described in the Appendix.

First, we conducted experiments to check whether the 15 debugger-detection techniques work in the Arm-Linux environment. The experimental results are presented in Table 1.

Next, experiments were conducted to test whether the proposed method automatically detects and patches the debugger-detection techniques. The experiments were conducted only for the debugger detection techniques that worked correctly in the Arm-Linux environment. In the experiment, the proposed method was applied to trace data obtained in the environment with and without gdb to determine whether it would automatically find the differences and perform a patch.

**Figure 5:**
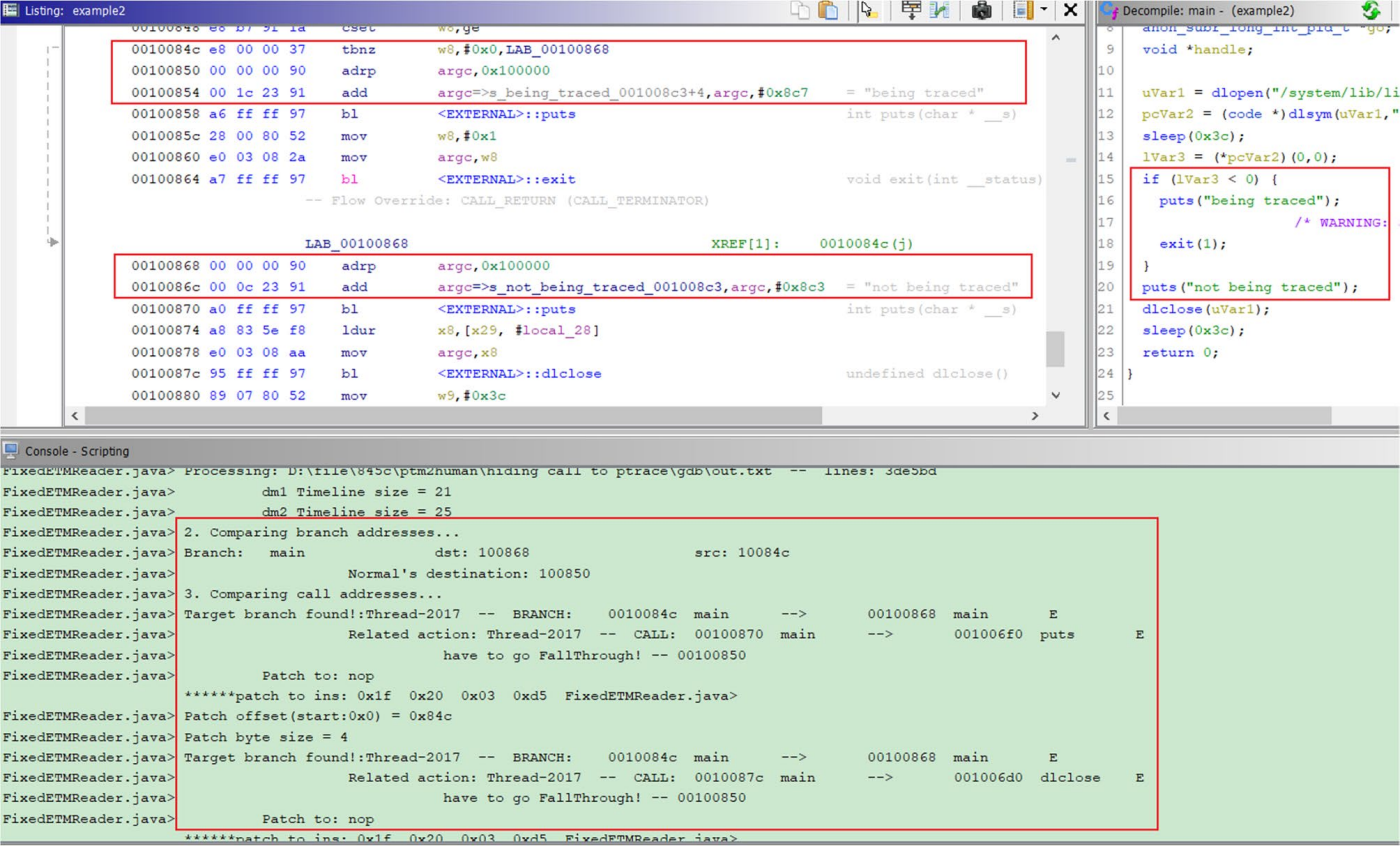
Example of detecting the debugger routine using the proposed scheme.

*Detection using ptrace(), hiding call to ptrace()* To detect these techniques, first, we should find API function calls regarding ptrace(). Subsequently, whether there is a fork in the trace is checked. In both techniques, the proposed method successfully identified the point at which a trace branch occurred and automatically performed the patch. Figure 5 shows the experimental results.

*Detection using SIGTRAP* This technique first registers a signal handler using signal() or sigaction() and then raises a signal (SIGTRAP). Hence, after calling these functions, a fork in the two traces indicates the presence of a debugger detection routine.

The proposed method determines the point at which the trace diverges and automatically performs patching. The patch point is the conditional jump instruction used to check whether the handler is executed because the handler is not executed within the debugging environment. This technique is used by Mirai malware, which is explained in the next subsection.

*Detection using /proc/$PPID/cmdline, /proc/$PPID/status, /proc/self/status* These techniques read specific files under the /proc directory to check the presence of debuggers. Hence, after reading the content of such files, a fork in the two traces indicates the presence of a debugger-detection routine.

These techniques detect a debugger by checking the execution status in the /proc directory, and our implementation successfully located the conditional branch instruction regarding debugger-detection and patched it correctly. For /proc/$PPID/cmdline and /proc/$PPID/status, if the gdb is attached during the execution of the test program, the presence of gdb cannot be detected. This is because when we attach gdb to the target process, the parent process of the target process is not gdb but the shell program.

*Detecting breakpoints, checking execution speed* The proposed scheme successfully detected a fork in two traces and found the debugger detection routine because our test program was very small (about less than 50 lines in C code), with the majority of the code related to debugger detection. Hence, in our test, there were no false-positive cases in which a trace fork appeared for other reasons. In general, there are diverse ways of measuring the execution speed or checking the memory contents for breakpoint instruction detection. We believe that fully automatic detection with no false positives is very difficult.

*Detection before main()* The proposed method found the point where the trace diverges and automatically performed patching. It also successfully found the conditional branch in the trace in a function called before main() and performed patching.

**Table 2:** Summary of experimental results for debugger detection techniques on the Arm-Linux system.

	AArch64-Linux	AArch64-Linux (attach)	Automatic detection	Automatic patching
Total	15	15	7	7
(Successful)	(9)	(7)	(7)	(7)

Table 2 summarizes the experimental results. Experiments were conducted using 15 debugger detection techniques. In the Arm-Linux environment, the debugger detection techniques worked correctly for 9 of the 15 cases when executing the target program under gdb. For attaching gdb, they worked correctly in 7 of the 15 cases in Arm-Linux environments.

The proposed scheme was tested on seven correctly working debugger detection techniques to determine whether it can automatically detect their presence by analyzing hardware trace logs and automatically patch them. In all seven cases, the proposed method accurately detected the debugger routines and automatically patched them to neutralize debugger detection.

### Case study: finding the debugger-detection routine in the Mirai sample

To verify the effectiveness of the proposed technique, we conducted experiments on real malware samples in Arm-Linux environments. First, experiments were conducted on Mirai bot, which is one of the most widely used Linux malware. The source code is provided in^13^.

Explanations of the overall structure, operations, and behaviors of Mirai are omitted due to space constraints, focusing only on the debugger detection routine. The bot code in Mirai malware, lines 63-68 of main.c, is as follows:
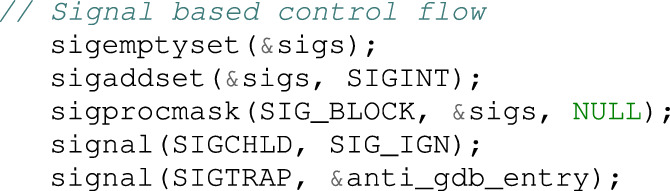


In the code above, when the signal (SIGTRAP, &anti_gdb_entry) function is called, the anti_gdb_entry() function is registered as a handler. This implies that, when the bot receives SIGTRAP, the anti_gdb_entry() function is executed. This is the debugger-detection technique using SIGTRAP, which was explained in the Background section. The anti_gdb_entry() function is in lines 353-356 of main.c.
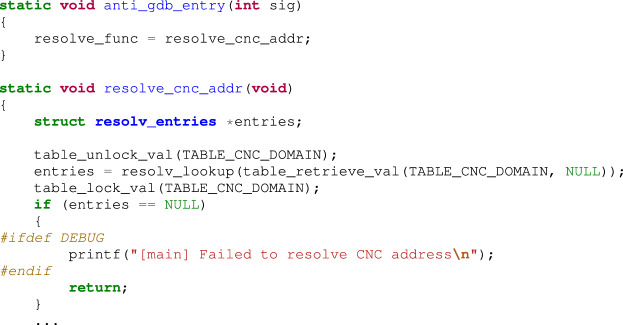


The anti_gdb_entry() function sets the resolve_func function pointer variable to the start address of resolv_cnc_addr(). This function calculates the IP address of the command-and-control (CnC) server, and the beginning of the function body is as described above.

Inspecting the code that generates the SIGTRAP signal in main.c, the signal is raised in line 114, as shown below (raise(SIGTRAP)). If this bot is compiled without the DEBUG flag, raise(SIGTRAP) is executed, which is located within the main() function of main.c. In short, at the beginning of the main() function, when starting a bot program, a signal handler is registered; a watchdog is set; and SIGTRAP is generated.
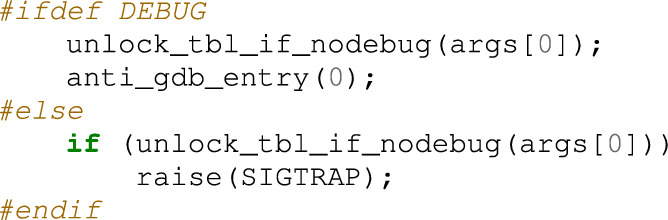


When SIGTRAP occurs, the registered handler anti_gdb_entry() function is called and the resolv_func function pointer is set to resolve_cnc_addr(). However, when running using a debugger such as gdb, the debugger catches SIGTRAP, and the anti_gdb_entry() function is not called, which implies that resolv_func variable has the initial setting value.

After generating SIGTRAP, the program connects to the CnC server using the code given below. At this point, _connection() is called to establish a connection with the CnC server.
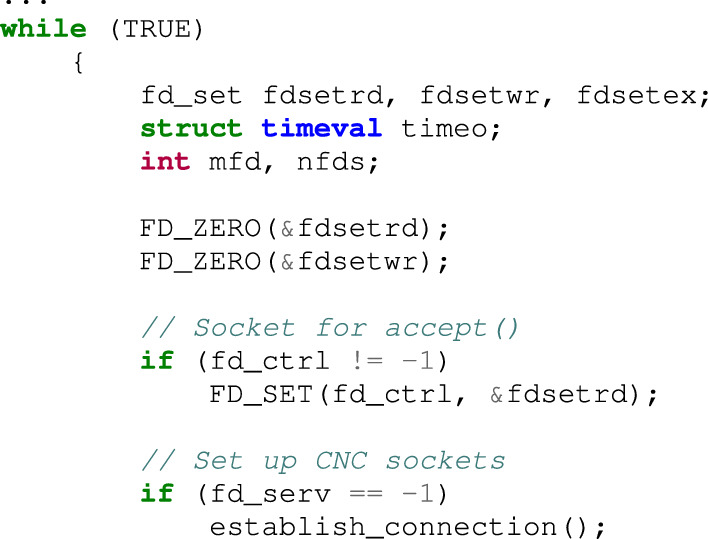


The contents of the establish_connection() function are as follows: Calling the reslov_func() function pointer is of primary importance in connecting to the CnC server. If the debugger is not connected, the resolv_func() function pointer points to the anti_gdb_entry() function in the signal handler, and the anti_gdb_entry() function is executed. Otherwise, in the debugging state, the address of the CnC server is not calculated accurately (because resolve_cnc_addr() is not called); therefore, no connection is established to the CnC server. Thus, the analyst cannot conduct a dynamic analysis with respect to the botnet related to the CnC server.
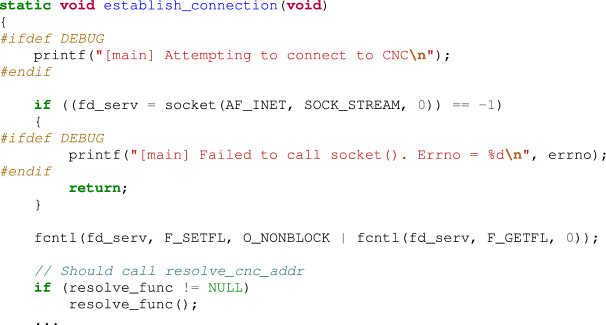

**Table 3:** Elapsed time for tracing, decoding, and analysis.

Execution without ETM trace	Execution with ETM trace	Decoding time	Analysis time
Mean (SD)	Mean (SD)	Mean (SD)	Mean (SD)
35.055	35.068	43.14	5.86
(0.014)	(0.068)	(0.58)	(0.365)

**Figure 6:**
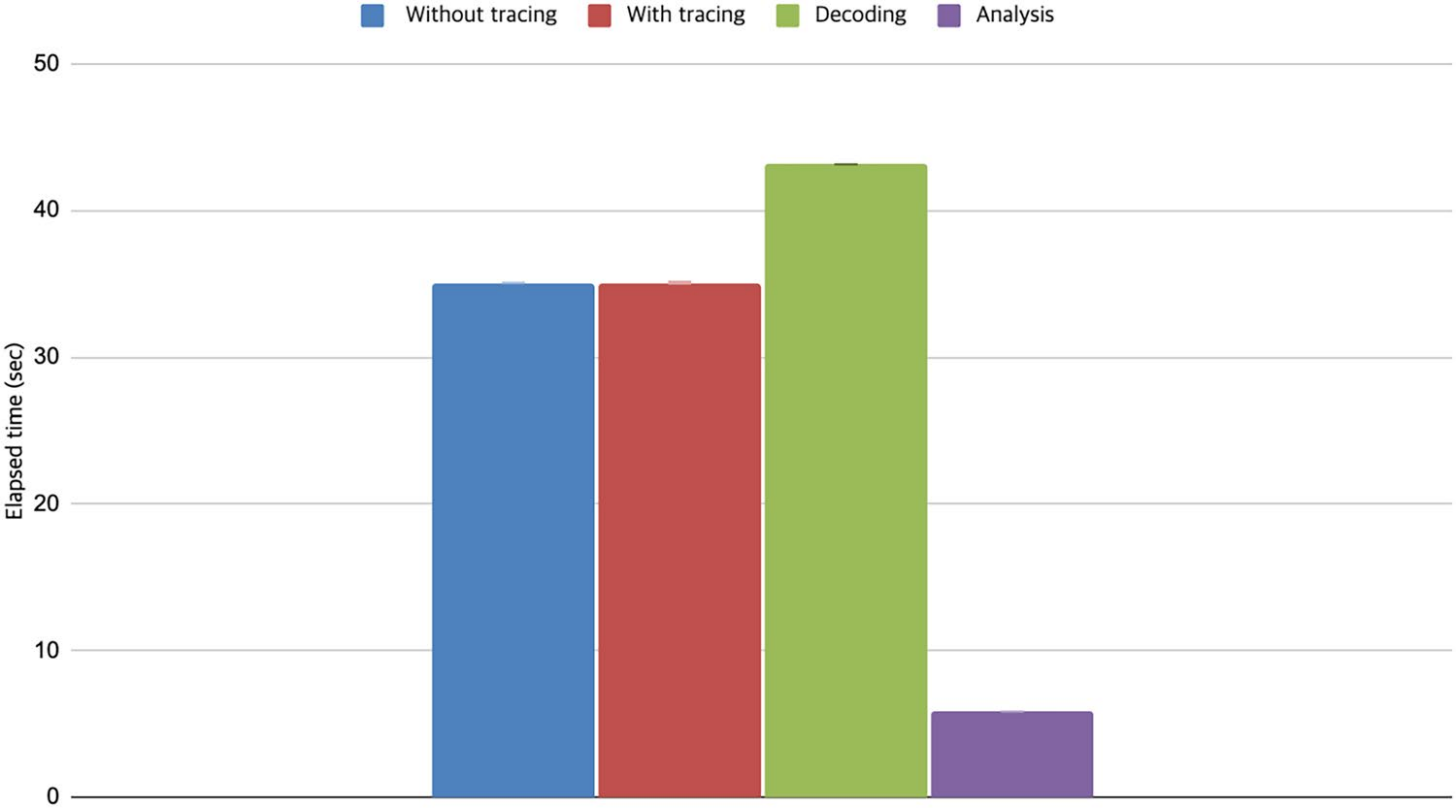
Elapsed time for tracing, decoding, and analysis.

Table 3 and Fig. 6 show the elapsed time in our experiments in obtaining the ETM trace, decoding the trace, and analyzing the decoded trace, which was repeated 20 times to obtain an average value. As shown in Table 3, the overhead for generating the ETM trace is negligible, at only 0.37%. The decoding process took approximately 43 s, which was the longest time. The analysis took only approximately 6 s, which is shorter than the decoding time.

Using the proposed technique, we automatically found differences between the trace executed with and without a debugger and accurately determined raise(SIGTRAP) as the branch point. Thus, the proposed technique can automatically and accurately detect the debugger detection technique used for the Mirai malware sample.

### Finding the debugger-detection routine in the real malware samples

Experiments were conducted on real malware binary samples obtained from the Internet. The samples were collected from VirusTotal.com and VirusShare.com. For the Mirai, Gafgyt, and Lightaidra families, we collected 378, 43, and 2 samples, respectively.

First, using disassemblers and decompilers, these samples were manually analyzed to check whether they contained debugger detection routines, as shown in Table 4. Of the 423 samples, 57 were manually determined to have debugger-detection routines.

**Table 4:** Malware samples and debugger-detection routines.

Malware family	# of samples	# of samples having debugger-detection routine (ptrace)	# of samples having debugger-detection routine (SIGTRAP)	# of samples having no debugger-detection routine	# of samples successfully executed	# of samples unsuccessfuly executed
Mirai	378	1	56	322^a^	71	307
Gafgyt	43	0	1	42	2	41
Lightaidra	2	0	0	2	0	2

^a^The reason why not 321 is because 1 sample contains both ptrace() and SIGTRAP routines.

Subsequently, experiments were conducted to verify that our implementation could automatically identify the debugger detection routine. Unfortunately, among all the samples, only 73 were successfully run in our experimental settings. Among them, only seven samples contained debugger detection routines, and 66 of them did not.

For these samples, there were no false positives, in the sense that our implementation did not falsely detect a debugger detection routine. In addition, for the samples that contained debugger detection, our implementation successfully identified the debugger-detection routine. We also manually checked 57 samples having debugger detection routines, confirming that all had the same logic for debugger detection. This implies that our implementation successfully detects debuggers, provided that they can be run in our experimental setting.

## Discussion

In conducting experiments, a total of 15 debugger detection techniques were employed. In the AArch64-Linux environment, the debugger detection techniques worked correctly in 9 of the 15 cases when executing the target program under gdb. Upon attaching gdb, 7 of the 15 cases worked correctly. For comparison, we repeated the experiments on x86-64-Linux systems with the relatively recent OS version (Linux 5.4, Ubuntu 20.04 LTS, CPU: i7-8700K, RAM: 48 GB) and obtained the same results. Based on this, we believe that a significant number of debugger techniques are currently not working because of changes over time in Linux, gcc, and gdb, making old techniques inapplicable to current versions.

Our implementation correctly detected the debugger-detection routine in all seven applicable cases (attach mode). Moreover, it automatically detects the debugger-detection routine in the Mirai sample.

An additional 423 malware samples were collected from virustotal.com and VirusShare.com, of which 57 were manually determined to have debugger detection routines. Of these samples, approximately 13.48% of the Linux malware samples had debugger-detection routines.

Unfortunately, only 73 of the samples could be successfully executed using our experimental setting. This is attributed to the fact that our experimental environment was AArch64-Linux whereas most of the collected samples were 32-bit. The AArch64-Linux system has limited compatibility with legacy 32-bit applications; for example, ARMv8 processors are backward-compatible only with ARMv7, and if the samples are compiled for ARM v5/v6, they cannot be run.

Among the 57 samples with debugger detection routines, seven were successfully executed in our experimental environment. Our implementation worked correctly for all seven samples having debugger detection routines.

We manually reviewed 57 samples with debugger detection routines, noting that all followed the same logic (ptrace, SIGTRAP). In addition, for SIGTRAP detection, we confirmed that all samples had the same structure as Mirai. This implies that our implementation would successfully detect them, provided that they could be run in our experimental setting.

Because we were only interested in the target application trace, the ETM was set to avoid tracing the kernel layer. Hence, tracing kernel-level daemons such as SOFT-IRQ or work-queue daemon, were not supported.

If the target program runs non-deterministically, the proposed approach may not work correctly. This is a well-known problem in dynamic analysis. Careful selection of the tracing points in conjunction with static analysis would improve performance, which we will pursue in future work.

## Related work

Related work is presented in three parts: hardware tracing, detection of anti-debugging techniques, and security-related research achievements using hardware functionalities.

In the first part, we discuss the CPU-level tracing functionality designed for debugging and optimizing systems and processes^14^, as in analyzing bugs or crashes and checking control flow integrity for safe execution. Diverse research efforts have been made to analyze crashes and bugs^15–20^. For control flow integrity, some approaches^21–23^ rely on Arm Coresight ETM whereas the others^24–26^ are based on Intel’s processor trace (PT). In this section, we focus on related work using hardware tracing for security, mainly for Arm-Linux systems. In addition, we briefly explain related open-source libraries.

CoreSight access library (CSAL)^27^ developed by Arm is a hardware trace-related software tool provided by Arm and operates in the Linux environment. It supports major reference boards, including the Juno Board, ST-Ericsson Snowball, and Arm TC2. To the best of our knowledge, for other reference boards (e.g., Dragonboard 410c or RB3 board), the hardware environment settings should be configured manually. Using CSAL, the hardware trace can be obtained from the Linux application.

OpenCSD^28^ is the CoreSight decoding library for Linux, developed by Linaro. Trace decoding is possible in conjunction with Perf. Decoding and displaying the trace log requires Perf and a Linux kernel with the CoreSight feature enabled. Currently, Debian Linux is supported.

In 2017, Malton^29^ was proposed at the 2017 USENIX Security Symposium. Malton is a scheme that analyzes malware in an Android environment. For analysis, Malton relies on DBI tool, Valgrind^30^. The disadvantage of this method is that it is a software-based approach, which implies that the execution speed is much slower than that of hardware-based approaches. In addition, it is sensitive to the version of the operating system and appears to be vulnerable to DBI detection techniques.

In 2017, Ninja^10^ was also proposed at the 2017 USENIX Security Symposium. Ninja allows transparent tracing and debugging using ETM. To the best of our understanding, Ninja supports only a single thread. It relies on special hardware, including a Juno board and DSTREAM. The trace buffer used is ETF rather than ETM and the buffer size is 64 KB, which implies that only very small traces are supported. Ninja’s advantage is that it can trace instructions, API calls, and memory reads/write using TrustZone and PMU.

Recently, a scheme called NCScope^31^ was presented at the ISSTA’22, which is an ETM-based HW-assisted analyzer for native code and is used to analyze Android applications. Using a kernel-tracing function called eBPF, malicious behavior of malware can be detected. Memory tracing is also possible using a hook in the kernel. NCScope relies on specialized software and hardware: DS-4 debugger, DSTREAM (to store large traces), and the Juno reference board. However, it appears to support only a single thread.

A scheme called Happer^32^ was presented at the 2021 Oakalnd’21 Conference. Happer is an automatic unpacking scheme for packed Android applications that employ ETM. It relies on DSTREAM and the Juno board, performs thread filtering rather than process filtering, and appears to support a single thread.

In 2020, HART^33^ was presented at ESORICS’20. HART is a hardware-assisted Linux kernel module-tracing scheme for the Arm environment. By modifying the kernel code, when the ETB buffer becomes full, the buffer is emptied using PMU. Thus, HART can record long traces at the kernel level.

Recently, JPortal^34^ was proposed as a hardware-trace-based JVM profiling tool. JPortal relies on Intel’s PT^1^ to obtain the trace log. Because in the JVM environment, the Java language is dynamically translated at runtime, interpreting the hardware tracing results is difficult. Nonetheless, precise and efficient control-flow tracing of JVM can be performed in JPortal. The experimental results show that JPortal has an overall accuracy of 80% for end-to-end control flow profiling with only a 4-16% runtime overhead.

In this section, we describe related studies for detecting anti-reversing techniques. In 2014, Smith et al. proposed REDIR^35^ for automated static detection of obfuscated anti-debugging techniques, translating the machine code to an intermediate representation (IR) using BAP and then using the rule engine in the expert system to automatically find anti-debugging techniques. They conducted experiments on 18 test cases and demonstrated that REDIR was 100% effective.

In 2017, Apate^36^ was proposed by Shi and Mirkovic as a framework for hiding debuggers using anti-debugging techniques. The authors designed and implemented diverse bypassing methods for anti-debugging techniques in 32-bit Windows. Apate was implemented as a WinDbg plug-in. The authors claimed that Apate outperformed other debugger-hiding schemes by a significant margin^36^.

In 2019, Park et al. proposed an automatic detection scheme for anti-debugging techniques in the Microsoft Windows Environment^37^. They focused on the Pin tool, which is a widely used DBI tool for dynamic analysis. Although Pin can automatically bypass some anti-debugging routines, it can be detected using complex routines. They designed and implemented a plug-in tool for Pin to automatically detect and bypass eight techniques.

Choi et al. proposed HybridEmu^38^, which is a dynamic analysis scheme for investigating the internal structure of malicious code in the Microsoft Windows 32-bit environment. Similar to xUnpack64^39^, HybridEmu can directly call or emulate various API functions in malware while emulating instructions using a 32-bit CPU simulator. However, it is designed only for 32-bit environments.

x64Unpack^39^ is a hybrid dynamic analysis tool for coping with diverse packers in the 64-bit Windows environment. It can find general unpacking routines and evade various anti-reverse engineering techniques. In addition, Choi et al.^39^ provided detailed analysis results for VMProtect, which is one of the strongest commercial packers.

Finally, we briefly describe security-related research achievements using hardware functionalities. Research efforts for devising attacks and defenses on computing systems use CPU functionalities, with many relying on performance counters.

In 2010, Tromer et al. explained the effective cache attacks against AES and their countermeasures by analyzing the state of the CPU memory cache^40^. In 2012, Xia et al. proposed a CFIMon system to detect attacks that violate the integrity of control flow, thereby demonstrating the feasibility of using performance counters to detect security vulnerabilities^25^.

Demme et al.^41^ discussed the possibility of using performance counters to detect malware, demonstrating that malware families can be detected on Android ARM and Intel Linux platforms. Yuan et al. proposed Eunomia, which uses a performance monitoring unit (PMU) to detect and identify security attacks^42^. They demonstrated that performance counters can accurately capture abnormal control flows caused by security vulnerabilities.

In 2014, Zhou et al. developed HDROP, a system that utilizes performance-monitoring counters to detect return-oriented programming attacks, demonstrating the application value of hardware performance counters in modern security defenses^43^.

In 2017, Patel, Sasan, and Homayoun analyzed the performance of various hardware-based malware detectors that rely on machine learning classifiers^44^. These classifiers use hardware performance counter information at runtime.

Recently, Carna et al. proposed a kernel-level infrastructure that uses system-wide performance counters to detect and mitigate side-channel attacks, to further demonstrate the importance of a performance-counter-based security strategy in a dynamic and complex system environment^45^.

## Conclusion

In this study, a novel scheme was proposed for finding debugger-detection routines using hardware tracing in an Arm-Linux system. The proposed scheme reconstructs the execution flow of the compiled binary code from trace data. In addition, it automatically identifies and patches the debugger-detection routine by comparing two traces (with and without the debugger). The proposed method was implemented using the Ghidra plug-in program, which is one of the most widely used disassemblers. To verify the effectiveness of the proposed scheme, we investigated 15 debugger-detection techniques in the Arm-Linux environment and tested whether they could be identified. The implementation was also tested for Mirai, one of the most well-known malware on Linux systems. The proposed scheme successfully identified the debugger detection routine. Additionally, we conducted experiments on 423 malware samples collected from the Internet, but only 73 could be successfully run in our experimental settings. Among them, only seven samples contained debugger detection routines and our implementation worked well for them. We believe that our approach will be helpful in future research on the tedious race between attackers and defenders.

### Supplementary Information

Supplementary Information.

## Data Availability

The malware data used in this study are available for non-commercial use at https://sites.google.com/view/haiminjin-tracinglinuxmalware/.
