# Supplementary material for: A practical approach for finding anti-debugging routines in the Arm-Linux using hardware tracing
Source: Sci Rep. 2024 Jun 26;14:14728. doi: 10.1038/s41598-024-65374-w (PMC11208535; doi:10.1038/s41598-024-65374-w)
Supplement: Supplementary file 1 — Supplementary Information. [file 41598_2024_65374_MOESM1_ESM.pdf]

Appendix

This section describes the implementation issues and experimental results of the 15 anti-debugging routines explained in the Background section. For Arm Linux, we used the DragonBoard 845c (db845c) in Linux Version 4.19. The host PC was a Linux-based Xeon E5-2680 with a 128 GB RAM workstation. The experimental environment for x86-64-Linux systems included: Linux 5.4, Ubuntu 20.04 LTS, CPU: i7-8700 K, and RAM: 48 GB.

Debugger detection using ptrace() A less than zero result of ptrace(PTRACE_TRACEME,0) implies that debugging is already in progress. Note that this function must only be called once. When called again, it unconditionally returns a negative number.

We conducted testing in x86-Linux and Arm-Linux environments, and were able to successfully detect the debugger in both environments.

Hiding call to ptrace()[1] If ptrace() is in a place that can be easily found in the source or binary code, analysts can easily find relevant anti-debugging routines. Here, we describe a method for hiding the ptrace() call using dynamic loading.

In our implementation, the function pointer 'go' was declared, and this pointer pointed to the ptrace() function through the dlsym() function. Finally, the function pointer was called in main() to execute the ptrace() function. The program running without the debugger outputs the message, "not being traced." whereas with gdb attached, it displays "being traced."

We conducted testing in x86-Linux and Arm-Linux environments and successfully detected the debugger in both environments.

Debugger detection using environment variables This technique detects debuggers by checking the environmental variables created while running the debugger. For example, when gdb is executed, it sets the environment variables 'COLUMNS' (terminal width length), 'LINES' (terminal height length), and '_=gdb_path' (the path of gdb). Hence, the presence of any of these three environmental variables, implies that the debugger was present during execution.

We wrote simple code and conducted the experiments. When running without gdb on x86-Ubuntu 20.04.1, no environmental variables related to the debugger were found. When running the test code in gdb 9.2, gdb created new environmental variables, which were detected. Similar results were obtained in the Arm-Linux environment.

Debugger detection using SIGILL SIGILL is a signal that occurs when an illegal instruction is executed and can be used to detect debuggers. In 80x86-Linux environments, the ud2 is an invalid opcode instruction and when executed, it generates SIGILL, and the corresponding handler is invoked. This is equivalent to the udf instruction in the Arm-Linux environment. If the debugger detects this signal and handles SIGILL, the original handler is not executed. Tools such as Pangu[2] use this technique for debugger detection.

Upon executing our code in x86-Ubuntu 20.04.1, a SIGILL signal was generated by the ud2 instruction, and the handler was called accordingly with 'No debugger' displayed. However, when running the target program under gdb, even if SIGILL occurs as intended in gdb due to ud2, the original handler is called and 'No debugger' is displayed, which means that this technique does not work properly in the 80x86-Linux system.

In the Arm-Linux environment, because the ud2 instruction is a machine code for 80x86, the ud2 instruction was changed to the equivalent udf in the Arm environment. No debuggers were detected in the experiment.

Debugger detection using SIGSEGV[2] SIGSEGV is a signal that occurs when there is a segmentation fault. This technique seeks to detect debuggers by invoking SIGSEGV and checking their behavior. In normal execution, when SIGSEGV occurs, the corresponding handler is invoked. However, in debugging environments, if the debugger intercepts SIGSEGV, the original handler may not be called. This technique was used to check for significant differences.

When running our implementation with gdb 9.2 on x86-Ubuntu 20.04.1, a SIGSEGV signal was generated by a segmentation fault, and our handler was called accordingly, outputting 'No debugger.' It seems that gdb detects SIGSEGV and passes this signal to the signal handler. In other words, we cannot use this technique to check for the presence of gdb in 80x86-Linux environments.

After modifying our code to cause a segmentation fault in the Arm-Linux environment, we cross-compiled the code and ran it on the db845c board. When executing our code in gdb 9.2, just as in 80x86-Linux, the debugger detected SIGSEGV and then our handler was executed displaying 'No debugger.' This implies that this technique does not work properly in Arm-Linux environments.

Debugger detection using SIGTRAP[2] In Linux, the SIGTRAP signal is used for debugging. When this signal occurs, the debugger intercepts the execution, and the original registered handler may not be executed.

Our implementation detects debuggers as follows. When executing our code without debuggers, because the handler is already registered using signal(), the handler is executed when SIGTRAP is raised. If the code is run in a debugger, the handler is not executed because the debugger intercepts the signal when SIGTRAP occurs.

In x86-Ubuntu 20.04.1 Linux, it was confirmed that our handler was not executed after the debugger acquired permission when SIGTRAP occurred as intended, and the debugger was detected. After cross-compiling our code, we ran it on the db845c

board. The same results as in the 80x86-Linux system were obtained, implying that this technique can detect the presence of the debugger in the Arm-Linux environment.

Debugger detection using file descriptors When a program is executed in Linux, the file descriptors 0 (standard input), 1 (standard output), and 2 (standard error) are fixed. Therefore, when a file is opened for the first time, 3 is assigned to the file descriptor for the new file in an ordinary case.

However, when opening a file for the first time in gdb, because more file descriptors have already been used for gdb-related internal work, a number greater than 3 is assigned to the file descriptor, which allows gdb to be detected.

However, in x86-64 Ubuntu version 20.04.1 and gdb version 9.2, with our implementation, gdb could not be detected. For example, 3 was assigned to the file descriptor even in the debugging environment. In addition, when the same code was run in the Arm-Linux environment, gdb was not detected.

Debugger detection using /proc/$PPID/cmdline /proc/$PPID/cmdline contains the contents of the command line used when executing the target process. We wrote code to detect gdb by opening /proc/$PPID/cmdline and checking whether this file contains 'gdb.' If /proc/$PPID/cmdline contains 'gdb', this indicates the presence of the debugger, and a message is output for debugger detection.

Running our implementation on x86-Ubuntu 20.04.1, when running our test code in gdb 9.2, gdb was detected as intended. However, the gdb was not detected if attached during the execution of the test program. This is because when the gdb is attached to the target process, the parent process of the target process is not gdb, but the shell program. The same results were obtained for Arm-Linux.

Debugger detection using /proc/$PPID/status The file /proc/$PPID/status contains diverse information on the execution status of the parent process. Our implementation accesses /proc/$PPID/status and checks the file for 'gdb' or 'ltrace' and further checks whether 'strace' is included. If these strings are included, the debugger is assumed to be running.

When running the code in x86-Ubuntu 20.04.1 in gdb 9.2, gdb was detected as intended and the message 'debugger detected' was output. However, the gdb could not be detected if it was attached during the execution of the test program. This is because when gdb is attached to the target process, the parent process of the target process is not gdb but the shell program. The same results were obtained for Arm-Linux.

Detecting breakpoints This technique detects the debugger by reading the opcode of the program and stops execution if it matches the opcode of the breakpoint used by the debugger. The opcode of the breakpoint instruction in x86-Linux environment is 0xCC or 0xCD, which can be checked in the code region. In the Arm environment it is 0xbebe (BKPT) in thumb mode or 0xe7f001f0 (UND) in 32-bit Arm mode. In AArch64, the opcode for BRK is 0xd4200000.

We wrote simple code and tested it in an x86-Linux environment. If executing the debugger by setting a breakpoint, our code successfully detected it. In the AArch64-Linux environment, we used 0xd4200000 to check BRK instructions. With gdb attached and a breakpoint, our code successfully detected the debugger. In other words, it was confirmed that this technique works properly in Arm-Linux.

Debugger detection using LD_PRELOAD If the LD_PRELOAD environment variable is set during runtime, the dynamic library is loaded and executed based on the environmental variable value. Detection becomes difficult when the debugger detection code is placed in custom library code and the library is loaded using LD_PRELOAD.

We wrote the ldpreload() function to check whether 'LD_PRELOAD' is among the program environmental variables. The ldpreload_custom_getenv() function calls the ldpreload() function to check, sets the value of 'LD_PRELOAD' to 'hello,' and then checks whether the value of this environment variable is 'hello' or was changed to other values (by gdb). A change indicates the presence of gdb.

We built the ptrace.so library with code that detects the presence of a debugger using the ptrace() function. The command in gdb was: 'set environment LD_PRELOAD=./ptrace.so.' When the ptrace() function is called, the ptrace() function of ptrace.so, not the ptrace() of the system library, is called, resulting in debugger detection.

When gdb is attached to this code, gdb cannot be detected even if 'LD_PRELOAD' environment variable is set in the command prompt of gdb. Therefore, this code can neither detect gdb in 80x86-Linux nor in Arm-Linux environments.

Debugger detection by checking /proc/self/status This technique checks the presence of debugging by examining the execution status of the program. To do so, the contents of /proc/self/status are checked. When a program changes from a non-debugging to a debugging state, the TracerPid entry in /proc/self/status changes from zero to a nonzero value, which is the process id of the debugger. Therefore, the TracerPid value in the status file can be checked to determine whether debugging is in progress.

First, we ran our code in the x86-Ubuntu 20.04.1 environment, whereby the UseProcStatus() function opens the /proc/self/status file and checks the TracerPid of the file. If TracerPid is not 0, it corresponds to the process ID of the debugger and indicates the presence of a debugger. In our experiments, the gdb was correctly detected. We ran the same code on the db845c board, thereby confirming that it correctly detects gdb in the Arm-Linux environment.

Debugger detection by checking /proc/self/fd This technique detects debuggers by checking the number of file descriptors

owned by the process. When the debugging process is initiated by gdb, it is created by forking the gdb process. Thus, when forking, the file descriptor owned by the parent process is inherited by the child process. Generally, gdb often opens multiple file descriptors, and they are also inherited in the target process. Specifically, the number of file descriptors owned by the process is /proc/self/fd, and this file can be checked. As this file is a directory file, each line of this file corresponds to the file information in this directory. For a new process, it contains six lines for six files (‘. /, ../, 0, 1, 2, and 3’). If the number of lines is greater than six, this indicates the presence of debuggers.

First, running our implementation in the x86-Ubuntu 20.04.1 environment, we opened the /proc/self/fd file and checked the d_name in the file. A value of ‘4’ or ‘5’ for dir->d_name indicates that debugging is underway. In our experiments, we could not detect the presence of debuggers in the x86-Linux system or the Arm Linux environment.

Detection before main() By hiding the debugger detection routine in the not-so-easily visible region in the code, the security can be increased. For example, if the “constructor” attribute in the C code is used, we can call the function before main(). For example, by declaring the function as follows, “void __attribute__((constructor)) before_main_function() {...},” we can call it before main()[1].

This code when being executed in x86-Ubuntu 20.04.1, works correctly and can detect the presence of the debugger just before the execution of main(). On the db845c board, we confirmed that this technique works correctly.

Detection by checking execution speed If the target program is executed using debuggers, the execution speed is slower than normal. An execution speed slower than the threshold indicates that the program is being debugged.

During implementation, when checking the execution speed, care should be taken when setting the threshold value. This code works when executing in x86-Ubuntu 20.04.1, correctly detecting the presence of the debugger. We confirmed that this technique also works correctly on the db845c board.

References

1. Baines, J. Programming linux anti-reversing techniques. https://leanpub.com/anti-reverse-engineering-linux (2016).

2. Voisin, J. Pangu: Toolkit to detect/crash/attack gnu debugging-related tools. https://github.com/jvoisin/pangu (2015).
